# Bioenergetic and Inflammatory Alterations in Regressed and Non-Regressed Patients with Autism Spectrum Disorder

**DOI:** 10.3390/ijms25158211

**Published:** 2024-07-27

**Authors:** Maria Gevezova, Zdravko Ivanov, Iliana Pacheva, Elena Timova, Maria Kazakova, Eleonora Kovacheva, Ivan Ivanov, Victoria Sarafian

**Affiliations:** 1 Department of Medical Biology, Medical University of Plovdiv, 4002 Plovdiv, Bulgaria; mariya.gevezova@mu-plovdiv.bg (M.G.); fm17008@mu-plovdiv.bg (Z.I.); mariya.kazakova@mu-plovdiv.bg (M.K.); eleonora.kovacheva@mu-plovdiv.bg (E.K.); 2 Research Institute at MU-Plovdiv, 4002 Plovdiv, Bulgaria; 3 Department of Pediatrics and Medical Genetics, Medical University of Plovdiv, 4002 Plovdiv, Bulgaria; iliyana.pacheva@mu-plovdiv.bg (I.P.); ivan.ivanov@mu-plovdiv.bg (I.I.); 4 Pediatrics Clinic, St. George University Hospital, 4002 Plovdiv, Bulgaria; elena.timova@mu-plovdiv.bg

**Keywords:** autism, metabolism, cytokines, COX-2, YKL-40, IL-1β, IL-9

## Abstract

Autism spectrum disorder (ASD) is associated with multiple physiological abnormalities. Current laboratory and clinical evidence most commonly report mitochondrial dysfunction, oxidative stress, and immunological imbalance in almost every cell type of the body. The present work aims to evaluate oxygen consumption rate (OCR), extracellular acidification rate (ECAR), and inflammation-related molecules such as Cyclooxygenase-2 (COX-2), chitinase 3-like protein 1 (YKL-40), Interleukin-1 beta (IL-1β), Interleukin-9 (IL-9) in ASD children with and without regression compared to healthy controls. Children with ASD (n = 56) and typically developing children (TDC, n = 12) aged 1.11 to 11 years were studied. Mitochondrial activity was examined in peripheral blood mononuclear cells (PBMCs) isolated from children with ASD and from the control group, using a metabolic analyzer. Gene and protein levels of IL-1β, IL-9, COX-2, and YKL-40 were investigated in parallel. Our results showed that PBMCs of the ASD subgroup of regressed patients (ASD R(+), n = 21) had a specific pattern of mitochondrial activity with significantly increased maximal respiration, respiratory spare capacity, and proton leak compared to the non-regressed group (ASD R(-), n = 35) and TDC. Furthermore, we found an imbalance in the studied proinflammatory molecules and increased levels in ASD R(-) proving the involvement of inflammatory changes. The results of this study provide new evidence for specific bioenergetic profiles of immune cells and elevated inflammation-related molecules in ASD. For the first time, data on a unique metabolic profile in ASD R(+) and its comparison with a random group of children of similar age and sex are provided. Our data show that mitochondrial dysfunction is more significant in ASD R(+), while in ASD R(-) inflammation is more pronounced. Probably, in the group without regression, immune mechanisms (immune dysregulation, leading to inflammation) begin initially, and at a later stage mitochondrial activity is also affected under exogenous factors. On the other hand, in the regressed group, the initial damage is in the mitochondria, and perhaps at a later stage immune dysfunction is involved.

## 1. Introduction

Autism spectrum disorder (ASD) is a heterogeneous group of neurodevelopmental disorders associated with the presence of social-communication deficits and restricted and repetitive behavior [1]. In recent decades, the number of diagnosed cases of ASD has been steadily increasing. The recent incidence in the USA is 1 in 36 children [2], with males 3 times more likely to develop the condition than females [3]. Although the exact etiology and pathogenesis of ASD are still unknown, it is believed to result from the integrated action of genetic and environmental factors that cause immune imbalance leading to oxidative stress and mitochondrial dysfunction [4,5,6]. Current laboratory and clinical evidence suggest that ASD involves systemic subclinical physiological disturbances not only in the central nervous system (CNS) [7]. This may explain the numerous concomitant diseases that affect energy-dependent cells such as neurons, and gastrointestinal and immune cells [8,9].

It is well established that children with ASD are a nonhomogeneous clinical group and show very different pathologies. Many studies have reported that up to 80% of individuals with ASD have unique changes in mitochondrial function other than those of classical mitochondrial disease [10,11]. Significant increases in electron transport chain (ETC) activity in multiple tissues [12,13,14], respiratory rate, and sensitivity to physiological stress have been detected [15,16]. In addition, 32% of children with ASD showed developmental regression in the second year of life [17], associated with loss of previous skills [18,19,20,21]. This was accompanied by a change in mitochondrial respiration and mitochondrial DNA (mtDNA) copy number [22]. A link between mitochondrial dysfunction, behavioral phenotype, and regression in autism has also been established [23,24]. These regressive changes usually occur after illness and immune system activation [25,26]. Therefore, various studies support the idea that a proinflammatory environment may play a role in the pathophysiology of ASD [27]. This is further supported by the fact that one of the main hallmarks associated with ASD is the observed cytokine imbalance [28], which is evidence of immune activation or immune-mediated inflammation. Increased levels of proinflammatory cytokines (IL-1β, IL-4, IL-5, IL-6, IL-8, IL-12p40, IL-12p70, IL-13, IL-17, TNF-α, and INF-γ) and reduced levels of anti-inflammatory molecules (TGF-β and IL-10) [6] are detected in postmortem brain tissue and cerebrospinal fluid samples, plasma, and mononuclear cell cultures [29]. The endophenotype of ASD associated with autoimmune dysregulation [30] and altered levels of immune mediators [31] has been suggested [32] to explain the observed regression in patients [33]. The immunological impact on the etiopathogenesis of ASD is supported by many studies because the brain can recognize the proinflammatory cytokines (IL-1β and IL-6) and tumor necrosis factor-alpha (TNF-α) as molecular signals of “disease” [34]. This proves that the nervous and immune systems are in constant two-way communication [35]. Major participants in both inflammatory and metabolic pathways, abundantly expressed in the CNS, are cyclooxygenase-1 and 2 (COX-1 and COX-2), which are key enzymes in the conversion of arachidonic acid into bioactive prostaglandins (PGs). COX-1 is constitutively expressed, while COX-2 is an inducible enzyme up-regulated under stress, inflammation, and physiological imbalance [36,37,38]. It is involved in the formation of lipid mediators such as PGs, which enhance cell signaling by cytokines, and induce chemokines and macrophages [39,40,41]. These changes activate the positive feedback loop that can mediate the transition from acute to chronic inflammation. Released PGs inhibit mitochondrial function [42,43] and NADH-ubiquinone reductase enzyme activity [44] and trigger reactive oxygen species (ROS) generation and apoptosis. This, in turn, causes a drop in membrane potential [45,46], blocks mitochondrial fission and deubiquitinating enzymes, and thus increases mitophagy [47]. All these alternations indicate that the immune imbalance affects not only the CNS but also mitochondrial activity and cell metabolism at different levels.

Another participant in the inflammatory diseases of the CNS is YKL-40, known as a chitinase 3-like protein 1 (CHI3L 1) or human cartilage glycoprotein 39 (HC-gp39). It is a chitin-binding lectin belonging to the glycosyl hydrolase family. The glycoprotein is expressed by different cell types, including macrophages, chondrocytes, neutrophils, and synovial fibroblasts. Its expression increases in neuroinflammatory conditions. YKL-40 has the capacity to regulate neuronal plasticity and regeneration. Bonneh-Barkay et al. (2010) suggests that the protein modulates neurotrophic factor-related changes in neuronal repair and regeneration [48]. Alterations in microglial function during critical developmental periods can cause disruptions in neuronal connections and synaptogenesis. The resulting neuronal damage leads to neurodevelopmental disorders (e.g., ASD and schizophrenia) and cognitive impairments in children [49]. The role of YKL-40 was recently investigated in the etiology of schizophrenia, and other neuroinflammatory diseases but not in patients with ASD [50,51]. In addition, the induction of YKL-40 extends beyond proinflammatory processes. Lee et al. (2019) revealed that the glycoprotein facilitates oxidative stress and chronic inflammation [52] and increases the expression of inducible nitric oxide synthase (iNOS), COX-2, TNF-α, IL-1β [52,53]. This highlights the key role of YKL-40 in the pathophysiology of inflammatory diseases and its potential as a diagnostic and prognostic biomarker. A number of studies have shown the importance of IL-9 and IL-1β in inflammatory diseases and ASD [30,54,55,56]. IL-9 is essential for the differentiation and activation of T cells in cases of autoimmune inflammation of the CNS [57]. It also increases the production of IgE and participates in allergic reactions that are part of the pathology of ASD [58]. In turn, IL-1 induces COX-2 expression and leukocyte infiltration preceding microglial morphological changes [59,60]. Another major neuromodulatory function of IL-1 is the inhibition of neurogenesis [61]. Therefore, these two cytokines seem to be crucial for the neuroinflammatory background in ASD and deserve special attention.

Even though ASD is a widespread and well-studied disorder, the underlying mechanisms behind its pathogenesis are still unknown or controversial. A further complication is the fact that the severity of symptoms and disease progression are unpredictable and vary from patient to patient. This necessitates a better understanding of bioenergetics and changes in the immune environment to stratify patients and guide the therapeutic approach.

Therefore, the present work aims to evaluate the cellular metabolic status and inflammation-related molecules such as COX-2, YKL-40, IL-1β, and IL-9 in ASD children with and without regression compared to healthy controls. Our hypothesis is that inflammation, mitochondrial dysfunction, and oxidative stress are interconnected and underlie the pathogenesis of ASD. Inflammation can lead to changes in mitochondrial activity, plasma, and transcriptional levels of proinflammatory and mitochondria-associated molecules. Oxidative stress, as a result of mitochondrial dysfunction and dysregulation of endogenous antioxidant mechanisms, inhibits mitophagy and results in the accumulation of damaged mitochondria, which contribute to the regression of the condition. In addition, inflammatory processes in the CNS lead to systemic release of cytokines and free radicals, which exacerbate neuronal damage.

The proinflammatory molecules of interest are poorly investigated in patients with/without regression. There are no published data on the correlation of these biomolecules with mitochondrial function and clinical manifestation of ASD in terms of regression. We present novel evidence for a unique metabolic profile in regressed children with ASD that may be useful for the development of new therapeutic strategies to counteract mitochondrial dysfunction and inflammation.

## 2. Results

### 2.1. Clinical Data

A target group of ASD children together with a control group of TDC were included in this study. Clinical data of both groups are presented in Table 1. TDC were matched to the ASD group in age and sex as closely as possible. A total of twenty-one of them had a history of regression (ASD R(+)) and 35 were without regression (ASD R(-)).

The patient and control groups were tested for routine hematological and biochemical parameters to confirm the absence of accompanying diseases.

### 2.2. Mitochondrial Activity

Bioenergetic parameters were measured using a Seahorse analyzer in 56 children with ASD and 12 healthy controls.

#### 2.2.1. Oxygen Consumption Rate (OCR) in Children with Regressive/Non-Regressive ASD and TDC

- Basel Respiration

Peripheral blood mononuclear cells (PBMCs) obtained from patients with ASD (with and without regression) and controls were found to have similar baseline oxygen consumption rate (OCR) (in pmol/min) (ASD R(+) = 28.99 ± 16.24 pmol/min, ASD R(-) = 26.62 ± 11.45 pmol/min; TDC 24.59 ± 7.83 pmol/min, *p* = 0.63), indicating a uniform beginning of the experiment.

- Maximal Respiration

**Figure 1:**
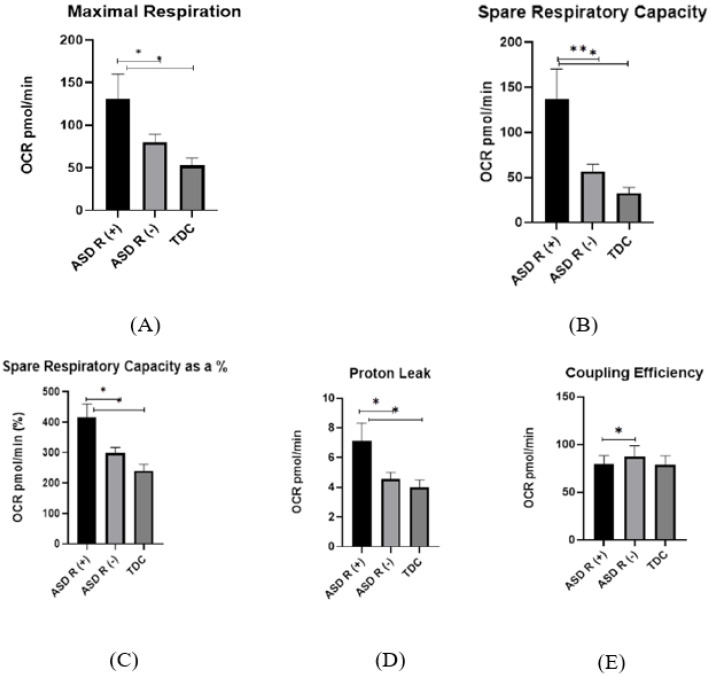

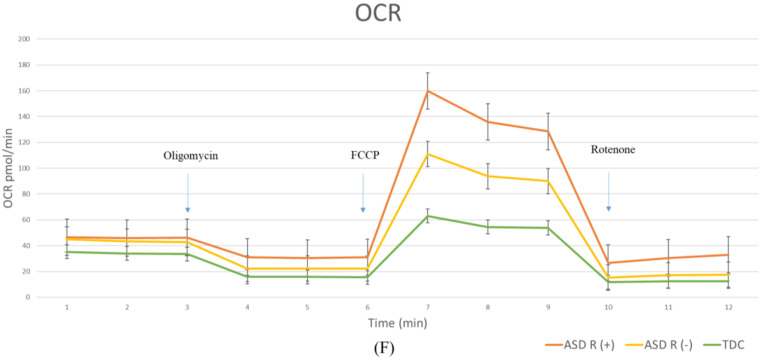
Respiratory metrics of PBMCs by Seahorse assay. (**A**–**E**) Bar graphs showing the differences in bioenergetics parameters between ASD R(+) and ASD R(-) patients and TDC, * *p* < 0.05, ** *p* < 0.01; (**F**) Effect of the used inhibitors (Oligomycin, FCCP, and Rotenone) on the oxidative function of isolated PBMCs.

The maximal respiration values in ASD R(+) cases (130.9 ± 97.05 pmol/min) were twofold higher than in control subjects (52.36 ± 22.36 pmol/min) (*p* = 0.02) (Figure 1A). A significant difference between ASD R(-) (79.88 ± 45.98 pmol/min) and ASD R(+) (*p* = 0.04) was detected (Figure 1).

- Spare Respiratory Capacity

A similar pattern was observed for spare respiratory capacity and spare respiratory capacity as a %. ASD R(+) patients had threefold higher respiratory capacity (137.1 ± 90.9 pmol/min) as compared to controls (32.5 ± 15.2 pmol/min, *p* = 0.03) (Figure 1B,C). The difference between ASD R(-) (65.73 ± 36.31 pmol/min) and ASD R(+) is statistically significant as well (*p* = 0.02). For spare respiratory capacity as a %, the statistical differences are similar. ASD R(+) showed value of 416 ± 42.61 pmol/min, for ASD R(-) it was 298 ± 19.06 pmol/min (ASD R(-)/TDC *p* = 0.01) and for TDC—240.2 ± 20.72 pmol/min (ASD R(+)/TDC *p* = 0.02) (Figure 1C).

- Proton Leak

There was also a significant difference in proton leakage between the investigated groups (ASD R(+) = 7.12 ± 3.96 pmol/min, ASD R(-) = 4.57 ± 2.16 pmol/min, (*p* = 0.03); ASD R(+) and TDC = 3.98 ± 1.39 pmol/min, *p* = 0.04), suggesting damage to the mitochondrial membrane in ASD R(+) patients compared to ASD R(-) and TDC (Figure 1D). It could be assumed that mitochondria still preserved the proton gradient across the membrane. In addition, we found decreased coupling efficiently in ASD R(+) (79.75 ± 8.6 pmol/min) compared to ASD R(-) (87.62 ± 2.18 pmol/min) (*p* = 0.04), but there was no statistical difference between ASD subgroups and TDC (Figure 1E).

- Non-Mitochondrial Respiration

No statistical difference was seen in non-mitochondrial respiration, indicating that ROS production by NADPH oxidase and other sources was not altered. There was also a significant difference in ATP production between the investigated groups. It is noteworthy that bioenergetic parameters were elevated in regressed patients, as demonstrated by the graphs shown. The differences between individuals with ASD and TDC depend on whether the ASD patient showed developmental regression. We found no statistical difference between the ASD R(-) and the control group.

- Coupling efficiency

Interestingly, the coupling efficiency was shown to be an important distinguishing parameter for both ASD subgroups. It was detected that ASD R(-) had a higher coupling effect compared to children with regression.

#### 2.2.2. Extracellular Acidification Rate (ECAR)

**Figure 2:**
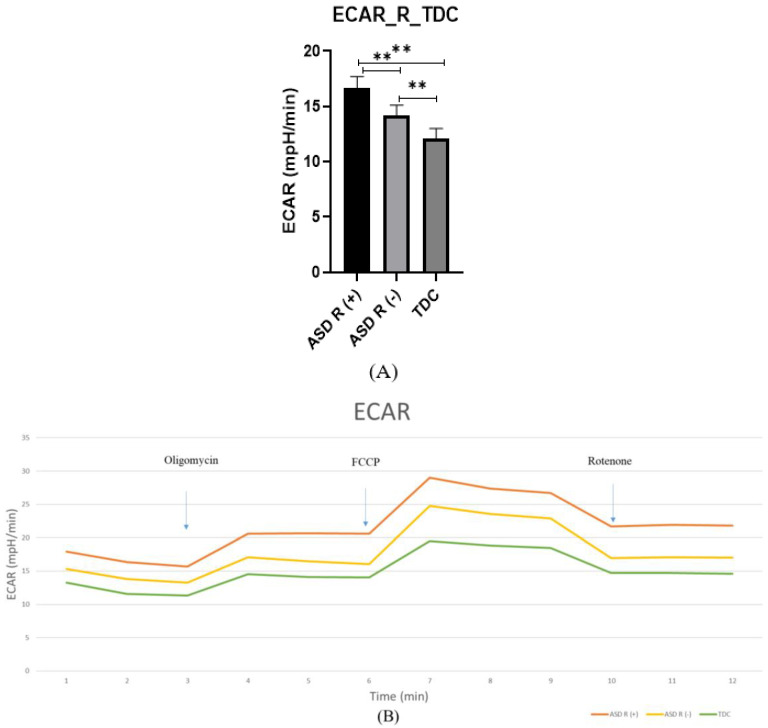
Extracellular acidification rate (ECAR) measured by Seahorse assay. (**A**) Bar graphs showing differences in glycolysis between ASD R(+), ASD/R(-), and TDC, ** *p* < 0.01; (**B**) Effect of the used inhibitors (Oligomycin, FCCP, and Rotenone) on the glycolytic activity of isolated PBMCs from the studied groups.

In addition to the observed differences in mitochondrial function, we also found enhanced glycolytic activity in the ASD group, which is measured by the extracellular acidification rate (ECAR). Interestingly, the regression group shows higher ECAR values compared to the other two studied groups. The ASD R(+) patients showed a greater increase in glycolysis (16.65 ± 0.42 mpH/min) compared to the ASD R(-) (14.14 ± 0.39 mpH/min) and control group (12.05 ± 0.38 mpH/min) (*p* = 0.003) (Figure 2A). These data support the results from our previous investigation proving the difference in glycolytic activity between patients and control groups. In this study, we provide additional evidence for a statistically significant difference between subgroups of patients with and without regression (*p* = 0.002) [24].

If we consider the changes in Figure 1F and Figure 2B after the introduction of the inhibitors, the PBMCs of both groups seem to compensate by switching between the Krebs cycle and glycolysis. This is evident even with the introduction of the first inhibitor, Oligomycin, which lowers OCR, i.e., mitochondrial respiration. In this case, the cellular response is related to an increase in ECAR as a compensatory mechanism for the loss of mitochondrial ATP.

### 2.3. Gene and Protein Expression of Proinflammatory Markers

The parallel gene and protein analysis showed increased levels of the proinflammatory markers COX-2, IL-1β, and up-regulation of YKL-40 mRNA.

- Gene expression of *COX-2*

**Figure 3:**
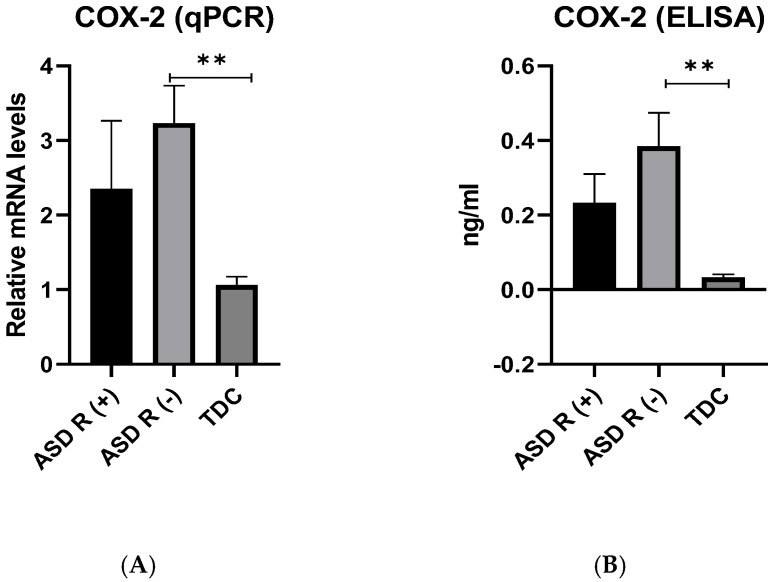
Gene (**A**) and protein (**B**) expression of COX-2 in patients with ASD R(+), ASD R(-) compared to TDC, ** *p* < 0.01.

Our results revealed that COX-2 expression levels were significantly higher in the patient subgroup (ASD R(-)) compared to controls: the expression of *COX-2* mRNA in ASD R(-) was 3.23 ± 0.50, while in ASD R(+) is 2.35 ± 0.91 and in healthy children 1.06 ± 0.11 (Figure 3A). The difference between patient subgroups with/without regression was not statistically significant, in contrast to ASD R(-) patients compared to the control group. An interesting observation was the fact that mRNA levels were significantly higher (*p* = 0.005) in the ASD R(-) compared to the control group.

- Protein expression of COX-2

The same pattern was observed for the protein level of COX-2 (Figure 3B). A higher concentration was detected in ASD R(-) (0.38 ± 0.08 ng/mL) compared to the control group (0.05 ± 0.005 ng/mL) with a statistically significant difference (*p* = 0.001). Again, no difference was observed between the ASD subgroups.

- Gene expression of *YKL-40*

**Figure 4:**
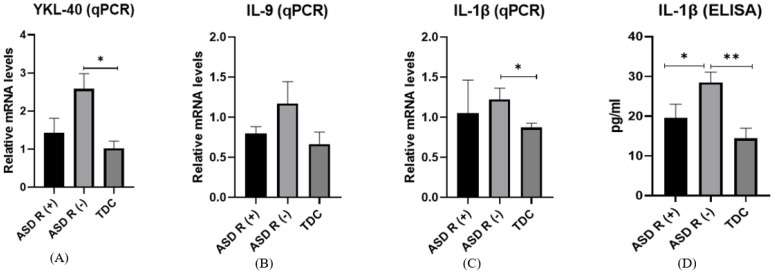
Gene expression of (**A**) YKL-40, (**B**) IL-9, (**C**) IL-1β, and (**D**) protein levels of IL-1β in ASD R(+), ASD R(-) and TDC * *p* < 0.05, ** *p* < 0.01.

The *YKL-40* mRNA values of patients with ASD R(-) and with ASD R(+) were 2.58 ± 0.39 and 1.43 ± 0.37, respectively (Figure 4A). A statistical difference (*p* = 0.04) between the patient group without regression and the controls (0.87 ± 0.14) was found.

- Protein expression of YKL-40

Surprisingly, the plasma concentration of the YKL-40 protein in all groups was below the detection limit of the kit (<23 ng/mL).

- Gene expression of *IL-9*

*IL-9* gene expression levels showed no statistical difference but tended to be higher in ASD R(-) children (1.17 ± 0.27) compared to ASD R(+) (0.79 ± 0.13) and TDC (0.63 ± 0.14) (Figure 4B).

- Protein expression of IL-9

Plasma concentrations of IL-9 in all study groups were also below the limit of detection (<1 ng/mL).

- Gene expression of *IL-1β*

The only cytokine that showed dysregulated expression in ASD subgroups was IL-1β. We found significantly higher *IL-1β* mRNA in ASD R(-) patients (1.22 ± 0.14) compared to healthy controls (0.87 ± 0.13) (*p* = 0.03). Again, there was no difference between the subgroups with and without regression (Figure 4C).

- Protein expression of IL-1β

The difference in IL-1β protein expression was also significant and followed the same trend in ASD R(-) (28.54 ± 2.52 pg/mL) compared to TDC (14.42 ± 2.6 pg/mL) (*p* = 0.04) (Figure 4D). Interestingly, we detected decreased protein levels in ASD R(+), with a concentration of 19.66 ± 3.32 pg/mL. The difference in protein levels was statistically significant (*p* = 0.01).

### 2.4. Correlation Analysis

Our study reports for the first time correlation data between mitochondrial parameters and levels of IL-1β in the ASD subgroup with regression (Table 2).

A positive association of mitochondrial parameters with the regression status in patients with ASD was confirmed. Regression correlated negatively with IL-1β protein levels. A moderate correlation between spare respiratory capacity as a % and regression was also detected. Regression correlated positively with proton leakage and negatively with coupling efficiency. These findings may reflect adaptive changes in mitochondrial function in response to chronic oxidative stress or the altered inflammatory environment [62,63].

## 3. Discussion

### 3.1. Novelty

Inflammation and mitochondrial dysfunction unite current theories regarding the pathogenesis of ASD. However, the available literature fragmentarily differentiates ASD subgroups according to the presence/absence of developmental regression. In this study, we observed a change in mitochondrial and glycolytic activity between patients with autism with/without regression. We also identified a distinct proinflammatory phenotype in ASD R(-) patients compared to ASD R(+) and TDC. In addition, we investigated the correlation between regression and the bioenergetic and inflammatory markers. These data are reported for the first time and demonstrate the important role of mitochondria in it and the unique metabolic phenotype of regressed patients. Frye et al. (2024) detected a similar phenotype between siblings of patients with ASD, but our results reveal that changes persist among a random (non-sibling) group of healthy children [64]. The differences in the proinflammatory markers in ASD R(-) are novel findings, which could indicate the leading role of inflammation in the early stages of this condition. In addition, we detected no statistically significant difference in the levels of the studied parameters between both genders in the patient and the control group.

### 3.2. PBMCs as a Model

PBMCs are a suitable model to track changes in both the periphery and the CNS because the phenotype of microglia in the CNS is similar to that of peripheral monocytes. Microglial cells produce pro- and anti-inflammatory cytokines and have immunocompetent functions. Activated immune cells secrete inflammatory and vasoactive substances, which further disrupt the integrity of the blood-brain barrier and thus facilitate the infiltration of immune cells into the CNS. Both animal studies and postmortem brain tissue samples from ASD individuals have proven microglial activation in different brain regions [65,66] and impaired blood-brain barrier [67,68]. In addition, there are numerous data about abnormalities outside the CNS associated with ASD-comorbidities such as disturbances/defects in the gastrointestinal, musculoskeletal, endocrine, immune, etc. systems [69,70,71]. All of them affect energy-dependent organs and systems. This has changed the view of ASD from a primary CNS disorder to a disease that affects multiple physiological systems. Many researchers explain the complex nature of ASD by the fact that the apparent mitochondrial dysfunction in patients may be related to chronic oxidative stress induced by immune-mediated inflammation [24,72,73]. A limitation in the usage of PBMCs is that they include a large spectrum of cell types and are not directly associated with the CNS. Although they have different composition, phenotype, and activation status than brain cells, PBMCs provide reliable non-invasive tools to study inflammation and mitochondrial function.

### 3.3. Mitochondrial Function in Children with Regressive/Non-Regressive ASD and TDC

Our results demonstrate that ASD R(+) PBMCs have a peculiar mitochondrial respiration pattern with basal respiration similar to that in ASD R(-) and TDC. Unlike the similarity observed in basal respiration, the results show, however, significantly increased maximal, spare respiratory capacity and proton leaks. A comparable trend was reported in a study on patients with ASD and a healthy sibling group by Frye et al. (2024) [64].

Mitochondrial respiration is considered as an indicator of the functional bioenergetic capacity of the organelle, and hence of the overall health of the cell [72]. The observed increase in respiratory parameters is a typical metabolic endophenotype in ASD R(+) [72]. These abnormalities in respiratory function render mitochondria vulnerable to physiological stress, which induces mitochondrial decompensation [21]. The observed phenotype is also consistent with previous studies in patients with autism and cell lines [22,24,74,75]. It is explained by a certain degree of stress in these organelles, leading to compensatory bioenergetic activation and chronic oxidative stress [15,24,72,73,75,76]. A recent study reports elevated bioenergetic parameters in regressed children and reveals that parents of children with ASD show atypical mitochondrial respiratory patterns similar to that of their children. The researchers assume that the abnormalities in mitochondrial function in this ASD group have a biological basis (like their parents) and are not intrinsic to maternally inherited mitochondria. A genetic or epigenetic component is suggested [64]. Therefore, the factors that regulate cell bioenergetics remain unknown or, at best, minimally defined. The elevated mitochondrial parameters could be explained by the cell’s behavior in trying to avoid the “ATP crisis”. When energy demands exceed energy supply, this leads to increased bioenergetics and allows the cell to survive [77,78].

Another explanation for the observed accelerated metabolic rate in ASD R(+) is the increased proton leakage compared to the other two studied groups. The leakage of protons through the inner membrane leads to a decrease in the gradient of the inner membrane, which in turn is associated with reduced ATP production. According to Jastroch et al. (2010), leakage of protons and electrons from the mitochondrial membrane has a major impact on mitochondrial coupling efficiency and reactive oxygen species production [79]. They found that inducible efflux through adenine nucleotide translocase (ANT) and uncoupling proteins (UCPs) can be activated by fatty acids, peroxide products, and superoxide. In our previous study, we reported a highly fatty acid-dependent ASD phenotype, which may also explain the observed increased proton leakage [5]. In addition, a rise in proton leak was recently found to be a key contributor to mitochondrial dysfunction in Fragile X syndrome mice [80]. This previous study also demonstrated that increased mitochondrial proton leak is associated with atypical synaptic growth. When correcting the leak, synaptic growth is restored. Thus, the proton leak can be directly related to abnormal synaptic function in the ASD group.

Interestingly, the two ASD subgroups (with and without regression) differ in coupling efficiency of mitochondrial respiration (*p* = 0.04). The indicator is increased in ASD R(-) and this can be explained by the ability of mitochondria to better regulate ROS production and oxidative capacity [81,82]. The higher level of coupling efficiency indicates a lack of functional alternation in the efficiency of oxidative oxidation in ASD R(-) and explains the absence of changes in mitochondrial function compared to the control group. In addition to the observed differences in mitochondrial parameters (maximal and spare respiratory capacity), we also found significantly increased glycolytic activity in the ASD group compared to TDC. This may be explained by the activation of cells and reprogramming of their metabolism, rendering them more glycolytic to meet the higher energy and biosynthetic needs [83]. Palmieri et al. (2020) suggest that metabolic switching may be a result rather than a cause of inflammation [84]. In addition, metabolites produced under glycolytic conditions can enhance the inflammatory response or lead to post-transcriptional and post-translational modifications [83].

In summary, the results reveal altered dynamics in mitochondrial parameters between ASD subgroups and controls. In ASD R(+), increased maximal, reserve respiratory capacity and proton leak are observed, which increases the susceptibility of organelles to stressors. Early identification of this mitochondrial overactivity could allow better protection of mitochondrial physiology in ASD R(+).

### 3.4. Inflammation

Chronic inflammation accompanies many mental disorders, including ASD [6,85,86,87]. It can be explained by the fact that the immune system and the nervous system are intricately interconnected. Both neurons and immune cells share many common molecules, such as cytokines, neurotrophins, and neurotransmitter receptors [24]. In conditions of chronic inflammation, present in many neuropsychiatric disorders, the blood-brain barrier is disrupted [88]. This allows the influx into the CNS of various immune components, cells, and neurotoxic debris that interfere with normal brain development and function [67].

To trace the relationship between inflammatory processes and mitochondrial activity, we investigated the gene and protein levels of COX-2, YKL-40, IL-1β, and IL-9.

### 3.5. COX-2

*COX-2* levels (mRNA and protein) were detected twofold higher in ASD compared to controls. Interestingly, we identified a correlation between *COX-2* mRNA and the bioenergetic parameters coupling efficiency and ATP. These findings suggest a link with the observed mitochondrial phenotype in ASD and support the reported compensatory bioenergetic activation due to inflammation [72]. *COX-2* is normally induced in the periphery in response to inflammation, but has constitutive expression in the brain as well, mainly in neurons [89,90]. It is important for normal neuronal function because COX2/PGE2-mediated signaling is involved in fundamental brain processes such as dendritic spine formation, synaptic plasticity, memory, and learning [89]. It is also associated with the high prevalence of seizures in autism [91]. In addition, several studies have demonstrated stimulation of COX-2 expression by NF-B activation following mitochondrial dysfunction in inflammatory pathologies [92], which supports the obtained statistical data.

### 3.6. YKL-40

Another novel biomarker molecule, YKL-40, is related to inflammatory response and is involved in the remodeling of the extracellular matrix. Accumulative evidence has highlighted its role in neuroinflammation and chronic systemic inflammation [53,93,94]. However, there are only a few studies on *YKL-40* gene or protein expression in patients with autism which show great variability in the results. Our data reveal that *YKL-40* mRNA levels are increased in ASD R(-) versus ASD R(+) and are nearly threefold higher compared to the TDC. Some researchers compared the transcriptome of the temporal cortex of postmortem brains from ASD subjects with age-matched healthy controls. They found a dramatic increase in the expression of immune system-related genes, one of which is CHI3L1 [95]. Consistent with our findings of increased levels in ASD R(-), Demirci, et al. observed a positive correlation between YKL-40 levels and ASD severity, suggesting that the glycoprotein has significant predictive value [96]. Numerous studies have found that CHI3L1 induces proinflammatory cytokines (IL-1β and IL-6), especially in some chronic inflammatory conditions [53,97]. This indicates the cooperative role of CHI3L1 with different molecules in modulating inflammatory responses and establishing feedback loops in various chronic inflammatory conditions.

Our previous results, as well as data from other studies, showed that YKL-40 levels increase with age and/or with inflammation [98]. Overexpression and secretion are observed mainly in diseases with systemic inflammation (systemic sclerosis, rheumatoid arthritis) or cancer progression [99,100,101]. Recently, a research group concluded that YKL-40 discriminates Alzheimer’s disease (AD) from controls and may predict the progression from the early preclinical to the late dementia stage. In genetic AD, YKL-40 increases decades before the clinical disease onset [102].

### 3.7. Il-1 and Il-9

Our study reported increased levels of *IL-1β* and *IL-9*, but only in ASD R(-) compared to ASD R(+) and controls. A negative correlation of *IL-1β* with regression was also detected. Similarly, Jyonouchi (2019) reported increased IL-1β-related signaling pathways affecting the nervous and immune systems [63]. When cytokine production is constant and excessive, it leads to the progression of chronic inflammatory diseases [103]. Therefore, cytokine profiling will reveal dysregulation of the immune system in ASD. It would be important to find out when these abnormalities start so that preventive treatment can be applied to counteract the changes. Thus, identifying mitochondrial and immune malfunction in the ASD group at an early age could help the protection of organelle physiology and the maintenance of cytokine-mediated homeostasis.

Frye et al. (2024) reported that siblings of children with regressed ASD were resistant to changes in mitochondrial function and suggested a genetic or epigenetic component in the development of abnormal mitochondrial respiration [64]. Perhaps, the explanation lies in the inflammatory state or the higher immune activation acting as an endogenous stressor. Yi-Chun Liao et al. (2023) investigated the effect of the proinflammatory environment on mitochondrial function and neuronal activity in hypothalamic neurons. It was found that spare respiratory capacity and maximal respiration were significantly compromised by the inflammatory environment [62]. The extent of mitochondrial alteration was highly dependent on the duration of the exposure to the inflammatory environment [62].

Based on our results, we can hypothesize that mitochondrial dysfunction and immune dysregulation have distinct roles in ASD subgroups and are not always interdependent. This may also explain the heterogeneity between individuals and the observed clinical symptoms. Our data show that mitochondrial dysfunction is more significant in ASD R(+), while in ASD R(-) inflammation is more pronounced. Probably, in the group without regression, immune mechanisms (immune dysregulation, leading to inflammation) begin initially, and at a later stage mitochondrial activity is also affected under exogenous factors. On the other hand, in the regressed group, the initial damage is in the mitochondria, and perhaps at a later stage immune dysfunction is involved.

Krakowiak et al. showed that circulating levels of IL-1β and IL-4 detected at birth were independently associated with ASD, with clinical symptoms occurring between 2 and 5 years of age [104]. A correlation with the severity of ASD was also observed, with IL-4 being associated with severe forms of ASD (according to the Autism Diagnostic Surveillance Scheme score) and IL-1β with mild ASD forms. Increased expression of IL-1β and IL-4 indicates prenatal immune abnormalities and thus supports a potential contribution to the pathogenesis of ASD [104]. Erbescu et al. (2022) discuss oxidative stress and mitochondrial function as participants in the proinflammatory immune-mediated pathways that influence brain development and function in ASD [104].

An important aspect for a better notion of ASD is the deeper investigation of changes in the levels of molecules related to inflammation and neurotrophic factors at different ages of patients with autism. More research is needed to improve the understanding of the heterogeneity in ASD, which in turn could facilitate treatment options [105].

### 3.8. Correlation between Metabolic State and Regression in ASD

In this study, we detected a relationship between increased metabolic activity and regression in the ASD R(+) subgroup. In a previous study on ASD PBMCs, we found that patients had significantly higher bioenergetic indices compared to controls. For the first time, after splitting the present ASD cohort into two subgroups, we report that ASD R(+) children show a phenotype distinct from that of ASD R(-) and TDC. Mitochondrial overactivity in patients has been found in ETC Complex I in muscles [106], fresh-frozen superior temporal gyrus [14], buccal tissue [107], and others. Therefore, we believe that the change in cellular bioenergetics may explain the loss of skills in the ASD R(+) subgroup since the brain is the most metabolically active organ in the body [108]. High mitochondrial activity may also explain the excessive free oxygen species and oxidative stress in ASD. Proton leakage reduces ROS production, oxidative stress, and further organelle damage. Singh et al. (2020) also support our data on the relationship between maximal respiratory capacity and symptomatology in patients with ASD [22]. Although increased levels of proinflammatory molecules were observed in our ASD group compared to controls, it is not clear why only ASD R(+) showed mitochondrial dysfunction. A possible explanation is that ASD R(-) is resistant to environmental stressors as a result of genetic or epigenetic components. Thus, several studies indicate that prenatal deficiencies of elements such as Zn and Mn are associated with abnormal mitochondrial function [109,110]. Therefore, further studies are needed to establish the dynamics of mitochondrial activity and levels of inflammatory markers in ASD.

We report essential two-way changes in the ASD patient subgroups influencing cellular vulnerability. Firstly, inflammatory molecules are elevated and secondly, mitochondrial dysfunction is detected. It is necessary to add that IL-1, besides being a pro-inflammatory cytokine, also has a role in tissue repair and angiogenesis [111]. This may explain the observed negative correlation between regression and cytokine levels in patients with autism.

### 3.9. Limitations of This Study

Major limitations of our study are the small number of healthy controls used the unequal number of age and gender-matched participants, as well as the usage of PBMCs as discussed previously in Section 3.2. Further research on a larger cohort or longitudinal studies testing the influence of age and gender is needed. Other limitations of this study are that syndromic ASD was excluded on a clinical basis, that not all possible genetic investigations were performed for the exclusion, that there was a lack of adaptive scale assessments, and that we relied on only the ADOS method for diagnosis of ASD.

## 4. Materials and Methods

### 4.1. Subjects

This study included Bulgarian children (n = 56) with idiopathic ASD and 12 typically developing children (TDC) aged 1.11 to 11 years. All patients with autism were referred for neurological evaluation (Table 1). Diagnosis of ASD was established according to DSM-5 after a detailed historical, physical, and neurological examination. Based on the clinical examination, we selected only patients with probable idiopathic ASD (with no facial dysmorphic features and no known congenital anomalies) trying to rule out a possible syndromic/complex ASD. There is a limitation in the etiology of ASD patients because a few patients had genetic investigations like cytogenetics, MLPA screening for most frequent microdeletions, and the FMR1 gene. We also excluded some patients suspicious of syndromic ASD, because of clinical features, but had not undergone all needed genetic investigations like WES, for example, or microarray CGH.

All of the children were examined physically, neurologically, and with neurodevelopmental assessment. Blood analyses were performed on ASD and TDC groups in the clinical laboratory at the St. George University Hospital.

Inclusion criteria for idiopathic patients with ASD were set as follows:

1. Age 1.9–11 years.
2. No previous intake of high-dose vitamins, mineral supplements, or immunomodulators in the past 3 months.
3. No acute illness, epileptic seizures, or medication in the last month.
4. No accompanying comorbidities.
5. Completed informed consent from the parents for participation in the study in accordance with the instructions of the University Ethics Committee.

The exclusion criteria included:

1. If according to clinical criteria, the patient corresponds to probable syndromic/complex/ASD (performed by a team of pediatricians—child neurologists) or probable syndromic/complex/ASD proven through genetic or imaging methods.
2. Chronic diseases such as infections, bronchial asthma, diabetes mellitus, etc.

The requirements for the control group of TDC were:

1. Healthy/not covering ASD according to DSM-5/children with normal intellectual development (good school performance), without chronic diseases such as asthma, diabetes, gastrointestinal disorders, etc.
2. No acute illness and intake of medicines, or nutritional supplements such as vitamins, minerals, or immunostimulants one month before the examination.
3. Completed informed consent from the parents for participation in the study in accordance with the instructions of the University Ethics Committee.

All patients with ASD were examined with the ADOS-2 [112] test to assess communication, social interaction, and play. The children were tested by a team of pediatric neurologists, psychologists, and child psychiatrists, as well as by a specialist certified in ADOS [112] assessment and DSM-5 [1]. The ASD cohort was further stratified into two subgroups: patients with regression (ASD R(+)) and those without regression (ASD R(-)). Patients with regression were determined according to the widely accepted term “regressive autism” (Pediatric Clinics of North America, 2007). The latter refers to children who have normal development until the age of 1 to 2 years, after which there is a loss of language, social interaction, and other developmental milestones (Pediatric Clinics of North America, 2007).

According to ADOS level evaluation, patients with ASD were distributed to their severity: as level 2 and 3 in 54, and level 1 in 2 patients who are non-regressive.

This study was approved by the Ethics Committee at the Medical University of Plovdiv (Protocol No C-05-2/10.04.2020).

### 4.2. Mitochondrial Activity

#### 4.2.1. Isolation of Peripheral Blood Mononuclear Cells (PBMCs)

Blood samples were collected in EDTA-Vacutainer monovettes (S-Monovette 2.6 mL, Z-Sarstedt) in compliance with all conditions for venipuncture.

PBMCs were isolated using a standard Pancoll gradient solution (Pan Biotech Cat # P04-60500) and a centrifugation procedure according to the manufacturer’s protocol. Patients’ and controls’ blood samples were mixed with phosphate-buffered saline (PBS) pH 7.4 (Gibco, Waltham, MA, USA; 10010023) and pipetted onto Pancoll in Falcon 15 mL sterile tubes. The samples were centrifuged for 30 min at 400× *g* with minimal acceleration and deceleration of the centrifuge. The buffy-coat layer of PBMCs was aspirated into a new 15-mL Falcon tube and washed with PBS. Centrifugation was performed at 300× *g* for 10 min with normal acceleration/deceleration settings. The last step was repeated twice, and the isolated cells were cultured in RPMI-1640 medium (Pan Biotech Cat № P04-22100) supplemented with 10% FBS, 1% penicillin/streptomycin in a cell culture incubator overnight at 37 °C, 5% CO_2_ and high humidity. After 24 h, the cells were washed with Seahorse RPMI (Seahorse XF RPMI medium, pH 7.4, 500 mL Cat. № 103576-100). Their number and viability were determined using a “LUNA” automated counter (Logos Biosystems, Anyang, Republic of Korea). PBMCs were plated in poly-D-lysine coated Seahorse assay microplates (Seahorse XFp FluxPak Cat. № 103022-100) at a final concentration of 2 × 10^5^. Normalization of the number of cells in each well was performed with a Corning Cell Counter (Cytosmart, Axion BioSystems, Atlanta, GA, USA).

#### 4.2.2. Mito Stress Test

Oxygen consumption rate (OCR) and extracellular acidification rate (ECAR) were determined via the Seahorse XF Cell Mito Stress Kit (Agilent). Cells were seeded at a density of 2 × 10^5^ cells in 8-well microplates in Seahorse XFp pH 7.4 basal medium. Using the inhibitor oligomycin (1.5 μM), ATP production and proton leak were measured. A second inhibitor, carbonyl cyanide-4-(trifluoromethoxy)-phenylhydrazone (FCCP) (2 μM), was then injected to assess maximal cellular respiration (respiratory capacity). The FCCP-stimulated OCR can be used to calculate spare respiratory capacity as a value or percentage (%). Spare respiratory capacity depends on maximal and basal respiration SRS = (maximal respiration) − (basal respiration) and SRS as % = (maximal respiration)/(basal respiration)*100. Finally, rotenone (0.5 μM), was applied to assess non-mitochondrial respiration. The inhibitors are used to interfere with different components of the respiratory chain and mitochondrial respiration, providing information on ATP production, proton leaks, and maximal respiration (basal respiration + spare capacity).

### 4.3. Gene Expression of COX-2, YKL-40, IL-1β, and IL-9

#### 4.3.1. Isolation of Total RNA from White Blood Cells

After centrifugation, plasma and blood cells were separated. Plasma was aliquoted and stored at −80 °C for the following analyses. Erythrocytes were lysed using ammonium chloride-based lysis buffer (NHCO_3_ 3.4 mM, NH_4_Cl 155 mM, EDTA 96.7 µM). RNA was isolated from white blood cells (within the pellet) with Trizol (Thermo Fisher Scientific, Waltham, MA, USA, Lot. No. 1559602), following the manufacturer’s instructions. After RNA extraction, samples were treated with the TURBO DNA-free kit (Thermo Fisher Scientific, Waltham, MA, USA, Lot. No. AM1907) to remove residual DNA. Extracted RNAs were quantified at 260/280 nm absorbance by NanoDrop Nucleic Acid Quantification (Thermo Fisher Scientific, Waltham, MA, USA).

#### 4.3.2. Reverse Transcription and qPCR

Two μg of total RNA was transcribed reversely by RevertAid H Minus First Strand cDNA Synthesis Kit (Thermo Fisher Scientific, Waltham, MA, USA, Cat. № 00648151) according to the manufacturer’s instructions. The resulting cDNA was used to quantify COX-2, YKL-40, IL-1β, and IL-9 expression and served as a template for amplification in a quantitative PCR reaction by Genaxon GreenMasterMix (2x) (Genaxon Bioscience GmbH, Ulm, Germany, Cat. № M3023.0500) following the manufacturer’s recommendations. The following specific primers were applied for RNA transcripts of COX-2 (Fw 5′-TCCTAGTCCTCATCGCCCTC-3, Rev 5′-AGATTAGTCCGCCGTAGTCG-3′), YKL-40 (Fw 5′-CTGCTCCAGTGCTGCTCT-3′, Rev 5′-TACAGAGGAAGCGGTCCAAGG-3′), IL-1β (Fw 5′-AGTGTCTGAAGCAGCCATGG-3′, Rev 5′-AGTCATCCTCATTGCCACTGT-3′) and IL-9 (Fw 5′-AGATCCAGCTTCCCAAGTGCC-3′, Rev 5′-TCTCACTGAAGCATGGTCTGGG-3′). The obtained expression values were normalized to the levels of the reference genes: GAPDH (Fw 5′-AGGTCCACCACTGACACGTTG-3′, Rw 5′-AGCTGAACGGGATGCTCACT-3′), ACTINβ (Fw 5′-AGTGTGACGTGGACATCCGGA-3′, Rev 5′-GCCAGGGCAGTGATCTCCTCCT-3′). 3′) and hUBC (Fw 5′-TCCTCAGGCAGAGGTTGATCTT-3′, Rev 5′-GGACCAAGTGCAGAGTGGACTCTT-3′) (Integrated DNA Technologies, Leuven, Belgium). qPCR reactions were performed on a Rotor-Gene Q 600 (Qiagen, Hamburg, Germany) using the comparative 2^−ΔΔCt^ method. All samples were analyzed in duplicate.

### 4.4. Detection of COX-2, YKL-40, IL-1β and IL-9 in Plasma by ELISA

Plasma levels for COX-2, YKL-40, IL-1β, and IL-9 were measured using specific individual ELISA kits following the manufacturer’s recommendations. Plasma samples from ASD and TDC cases were analyzed with the following kits: COX-2 (Human PTGS2/COX-2 (Prostaglandin Endoperoxide Synthase 2) ELISA Kit; Cat. № E-EL-H1846, Elabscience Houston, TX, USA), YKL-40 (YKL-40 Human ELISA Kit, Microvue YKL-40 Cat. № 8020), IL-1β (LEGEND MAX™ Human IL-1β ELISA Kit; Cat. № 437007) and IL-9 (LEGEND MAX™ Human IL-9 ELISA Kit; Cat. № 434707). All samples were measured in duplicate. The assays employ a sandwich-based ELISA method by absorbance measurement at 450 nm wavelength on a TECAN SUNRISE reader. Data were then averaged for each sample and run for statistical analysis.

### 4.5. Statistical Analyses

Pre-processing of raw OCR and ECAR values was done using the Seahorse XF Mito Stress report generator Wave (available at https://www.agilent.com/). Statistical analyses were performed with GraphPad Prism 8. Data were assessed for normality by a Shapiro-Wilk test together with a quantile-quantile (QQ) plot. Differences between normally distributed variables were evaluated for significance by Welch’s *t*-test for independent samples or a paired *t*-test for dependent samples. For non-normal data, the non-parametric Mann-Whitney assessment was preferred. The significance threshold was set at a *p*-value < 0.05. Correlation analysis was performed using Pearson correlation. Results are presented as means ± SD.

## 5. Conclusions

The results of this study provide new evidence for elevated proinflammatory molecules in a subset of patients with ASD without regression compared to PBMCs of ASD children with developmental regression. A well-defined metabolic profile in ASD R(+) and its comparison with a random group of age- and sex-matched children is reported. These data may allow a better understanding of the pathogenic mechanisms of ASD, patient stratification, and its relationship with metabolism. In addition, we found that the levels of proinflammatory molecules depend on the presence or absence of regression among patients. The view that immunometabolism is a key player in autism opens new possibilities for therapy. An understanding of the pathways related to immune and mitochondrial regulation can help the development of new therapeutic strategies to counteract inflammatory and bioenergetic abnormalities in ASD.

## Figures and Tables

**Table 1:** Clinical data.

Variables	ASD R(+)	ASD R(-)	TDC
Number of patients	21	35	12
Age (mean)	3.05 ± 1.4	4.46 ± 2.5	7.63 ± 3.28
Sex M/F	19/2	28/7	8/4
Normal intelligence			IQ ≥ 70
IQ/DQ	50.06 ± 10.73	53.6 ± 14.05	not measured.
ADOS total points	27.53 ± 4.41	24.59 ± 5.59	not measured
Communication pts (ADOS)	6.23 ± 1.78	5.62 ± 1.88	not measured
Social Interaction pts (ADOS)	14.92 ± 3.45	13.48 ± 3.32	not measured
Repetitive Behavior pts (ADOS)	6.38 ± 1.27	5.74 ± 1.87	not measured.

**Table 2:** Correlation of the bioenergetic parameters, levels of IL-1β in ASD R(-).

Parameters		r
Regression	Spare respiratory capacity	0.499
	Spare respiratory capacity (%)	0.469
	Coupling efficiency	−0.343
	Proton leak	0.535
	IL-1β	−0.326

## Data Availability

Data will be made available on request.
